# Natural Sweetness and Bioactivity: The Cardiovascular Promise of Fruits

**DOI:** 10.3390/nu17213417

**Published:** 2025-10-30

**Authors:** Aleksandra Fałczyńska, Ewa Miller-Kasprzak, Dawid Rosiejka, Joanna Michałowska, Wiktoria Błażejewska, Adela Bogdańska, Paweł Bogdański

**Affiliations:** 1 Department of Obesity Treatment, Metabolic Disorders and Clinical Dietetics, Poznan University of Medical Sciences, 61-701 Poznan, Poland; 2 Medical Education, Wroclaw Medical University, 50-367 Wroclaw, Poland

**Keywords:** sugar, fruits, cardiovascular health, plant bioactive compounds

## Abstract

Cardiovascular diseases (CVDs) remain the leading cause of mortality worldwide. Their prevalence is expected to rise with demographic shifts and increasing obesity rates. Excessive sugar consumption, especially from added sugars and sugar-sweetened beverages (SSBs), is a major modifiable risk factor of CVDs. It contributes to obesity, metabolic disorders, type 2 diabetes, and cardiovascular disease. High-sugar diets affect metabolic and cardiovascular health. They also contribute to neurobehavioral dysfunction by influencing the brain’s reward pathways, promoting hedonic eating, and reinforcing dependence on sweet taste. Fruits, a natural source of sweet-tasting compounds, are widely considered healthier than processed sweets. Epidemiological evidence shows a protective link between fruit consumption and lower risk of acute cardiovascular events like stroke and coronary heart disease. This benefit is largely due to bioactive compounds in fruits, such as fiber, polyphenols, and micronutrients. Based on current evidence, fruits can satisfy sweet cravings. In this paper, we will discuss the potential of fruits as an alternative to added sugars, emphasizing their beneficial effects on metabolic and cardiovascular health.

## 1. Introduction

Cardiovascular diseases (CVDs) remain a major global health challenge. In 2021, mortality from CVDs accounted for 26.8% of all deaths, and from 1990 to 2021, the prevalence rate of CVDs increased by 0.88% [1]. In the United States, CVDs are currently the leading cause of death and account for 17% of total national health expenditure [2]. Given ongoing demographic shifts, the prevalence of CVDs is expected to rise further. This will likely lead to increased healthcare costs [2,3]. Risk factors include hypertension, dyslipidemia, hyperglycemia, overweight and obesity, smoking, physical inactivity, and unhealthy diets [4]. Obesity is a growing global health issue, affecting about 13% of the world’s population. It is now established that too much caloric intake, especially from sugar-rich diets and sedentary lifestyles, significantly contributes to obesity [4,5].

Excessive sugar consumption has been identified as a significant risk factor in the development of obesity, metabolic disorders, type 2 diabetes, cardiovascular disease, certain cancers, depression, and cognitive dysfunction. “Added sugars” are defined as sugars that are added in food preparation or manufacturing, such as glucose, fructose, sucrose (a disaccharide composed of two monosaccharides, glucose and fructose linked by a glycosidic bond), and hydrogenated starch hydrolysates (high-fructose corn syrup-HFCS). According to the World Health Organization (WHO) and the Scientific Advisory Committee on Nutrition (SACN), “free sugars” are all sugars that are naturally present in honey, fruit juices, and syrups and generally not considered to include sugars found within the cellular structure of foods. During the second half of the 20th century, due to the belief that dietary fat was the main contributor to CVDs and obesity, fat consumption declined while intake of carbohydrates, particularly refined sugars, increased. For example, the consumption of high-fructose corn syrup rose to 42% by 2000 from 1% in the 1970s. By 1999, per capita availability of added sugars and sweeteners in the United States exceeded 69 kg annually, coinciding with a rise in CVDs and metabolic diseases [5]. Sugar-sweetened beverages (SSBs) are the largest single source of added sugars in the diet. A typical 355 millilitre (mL) serving of soda contains 35–37.5 g of sugar and approximately 140–150 kilocalories (kcal) in total. Epidemiological evidence links excessive intake of SSBs and risk of metabolic syndrome, a precursor for cardiometabolic diseases, suggesting adiposity and dyslipidemia as partial mediators [6].

A high-sugar diet, in addition to contributing to metabolic disturbances, may also be connected to neurobehavioral impairments, resulting in changes in cognitive functions. Sugar influences the brain’s dopamine system in ways similar to cocaine addiction [7]. This activity is often discussed in the context of sweet taste perception, food reward, and binge-eating behaviour [8]. The sensory and rewarding properties of sugar—especially its sweetness—are considered major factors contributing to overconsumption [9]. Biological, sensory, cognitive, and social factors all shape sugar intake [10]. These interacting mechanisms help explain both the widespread appeal of sugar and the challenges associated with its consumption [11,12]. Finding alternatives to sweets is likely necessary to avoid their harmful behavioural effects [13].

Fruit, as a natural source of sugars, is often perceived as a healthier alternative to sweets. A protective association has been observed between higher fruit consumption and a reduced risk of acute cardiovascular events, such as stroke and coronary heart disease. Their cardioprotective properties are attributed to the content of specific nutrients responsible for the regulation of metabolic processes [14]. Notably, Scheffers et al. have shown that pure fruit juice, when consumed in moderation, may serve as a preferable substitute for SSBs and is associated with a lower risk of cardiometabolic disorders. This suggests that fruits can be both a sweet, healthy remedy for satisfying sugar cravings and a natural means of reducing cardiometabolic risk [15].

## 2. Biology of Sweet Taste Perception

Individual dietary choices are conditioned by heterogeneous determinants such as culture, society, psychology, genetics, or taste [16,17]. Presently, there is an ongoing scientific discourse regarding whether high sugar consumption is linked to a preference for sweet taste [10]. Sweet food is one of the basic taste modalities and, in general, is perceived as harmless and rewarding. Particularly, in the context of evolutionary biology, this may have indicated a nutritional and survival advantage of the meal [8]. Nowadays, where sugars are easily accessible, this innate serves as a problem and might lead to problems with overconsumption [18]. The complexity of mechanisms by which refined sugars affect the cardiovascular system is undoubtedly significant for human health. Many of these adverse processes occur gradually and are the consequences of chronic excessive intake of sugars. This leads to the question: why is sugar still consumed in large quantities despite the knowledge of its harmful effects? Biological and neurobiological mechanisms involving sweet taste perception and the dopaminergic reward system might be an answer to this phenomenon [19].

### 2.1. Sweet Taste Receptors

The gustatory system, which is responsible for taste perception, consists of taste cells organised in the frontal, lateral, and anterior parts of the tongue. Taste buds are onion-shaped structures consisting of 50–100 cells; their stimulation induces the release of compounds responsible for activating cranial nerves such as the facial, glossopharyngeal, and vagus nerves [20].

Sweet taste receptors are overall T1R2/T1R3 heterodimers, belonging to the G-protein coupled receptors family (GCPRs) [21]. Their expression was detected not only on the tongue, but also in the gastrointestinal system, especially on enteroendocrine cells, where they are responsible for glucose metabolism and the secretion of gastrointestinal hormones [22,23]. These receptors are also expressed in the pancreas, adipose tissue, and brain, where they are involved in the regulation of insulin secretion and satiety [21]. The T1R receptor is a complex protein composed of a large extracellular venus flytrap domain (VFD), a cysteine-rich domain (CRD), and a seven-transmembrane α-helical domain (TMD). Various ligands, including natural sugars, artificial sweeteners, and sweet-tasting proteins, can bind to distinct domains of the receptor. Moreover, each subdomain is capable of functioning independently in ligand recognition and signal transduction [20,21,24].

Detection of sweet-tasting molecules initiates an intracellular signalling cascade via a G-protein activation, stimulating phospholipase Cβ2 (PLCβ2) and producing inositol 1,4,5-trisphosphate (IP_3_). This triggers the release of Ca^2+^ and depolarization via the opening of transient receptor potential channel M4 (TRPM4) and transient receptor potential channel M5 (TRPM5) [20,21]. Adenosine triphosphate (ATP) is then released through calcium homeostasis modulator 1/calcium homeostasis modulator 3 channels (CALHM1/CALHM3), acting as a neurotransmitter that activates purinergic receptor P2X, ligand-gated ion channel, 2 (P2X2) and purinergic receptor P2X, ligand-gated ion channel, 3 (P2X3) on afferent sensory neurons [20]. The signal is transmitted to the nucleus of the solitary tract (NST) and subsequently relayed to higher brain regions, including the thalamus, amygdala, and hypothalamus [23].

Sweet taste may also be mediated differently from T1R-independent mechanisms—for example, by sodium-glucose cotransporter 1 (SGLT1) and glucose transporter type 4 (GLUT4). On the tongue, SGLT1 and GLUT4 are responsible for transporting glucose into sweet-sensing cells. Their selective activation by natural sugars suggests the ability to differentiate caloric sugars from non-nutritive sweeteners [21]. Tan et al. have shown in their study that SGLT1 acts as a conduit between the gut and the vagal nerve, taking part in the transduction of taste signalling to the brain. Authors demonstrated that other substances like galactose and the glucose analogue 3-O-Methyl-D-glucose (3-OMG19), and both substrates for SGLT-1, can also activate glucose-responsive vagal neurons. In contrast, other caloric sugars, such as fructose and mannose, elicit only osmolarity responses and do not drive a behavioural preference. Furthermore, authors used flozine, an SGLT-1 inhibitor, to demonstrate loss of glucose responses following intestinal application of this substance, which confirmed that SGLT-1 is an essential part of the gut-brain axis [22].

### 2.2. The Natural Drive for Sweetness

The brain serves as a significant element regulating food intake and shaping the behavioural aspects of the reward-related characteristic of food, as the main motivation of the feeding pattern [7]. However, recent studies draw attention not only to survival aspects, but also to modulating aspects of brain activation, especially in relation to the consumption of sugar and fat [25].

#### 2.2.1. Sugar Intake Regulation

Sugar intake is managed on the neurohormonal level by multiple interactions, especially via opioid, dopaminergic, and cholinergic signalling pathways. The glucoregulation is the process that maintains blood sugar levels by pancreatic hormones as insulin and glucagon [23]. Peptide hormones, including ghrelin and leptin, regulate the activity of the hypothalamic melanocortin system in the area of the arcuate nucleus (ARC) via the precursor of the peptide pro-opiomelanocortin (POMC) and CART (cocaine and amphetamine-regulated transcript), responsible for reducing food intake. Another pair of neurotransmitters controlled by peptide hormones are neuropeptide Y (NPY) and the melanocortin 4 receptor (MC4R); the antagonist agouti-associated protein (AgRP) stimulates appetite [20,23,26]. A further circuit involved in the process of food intake is connected with triggering positive sensations during consumption with GABA-ergic (gamma-aminobutyric acid) receptors and the lateral hypothalamus (LH) [26].

Stress-like reaction could also serve as a signal for food intake—it stimulates the secretion of cortisol, responsible for regulating metabolism of glucose, fat, appetite, and weight control [23]. Moreover, Klenowski et al. found that the brain’s answer to stress or anxiety, caused by increased levels of cortisol, may promote a preference for highly palatable food enriched in sugar, which activates the reward cycle in the brain [23,27].

#### 2.2.2. Reward Signaling System

There might be a great connection between dopamine, the reward-signalling system, and energy homeostasis. Dopamine is a neurotransmitter responsible for the process of neuronal learning and motivation—it affects behavioural responses in relation to rewards. Dopamine neurons are located in the ventral tegmental area (VTA), containing receptors that can be activated by ghrelin, leptin, insulin, and glucagon-like peptide-1 (GLP-1) [26].

**Figure 1:**
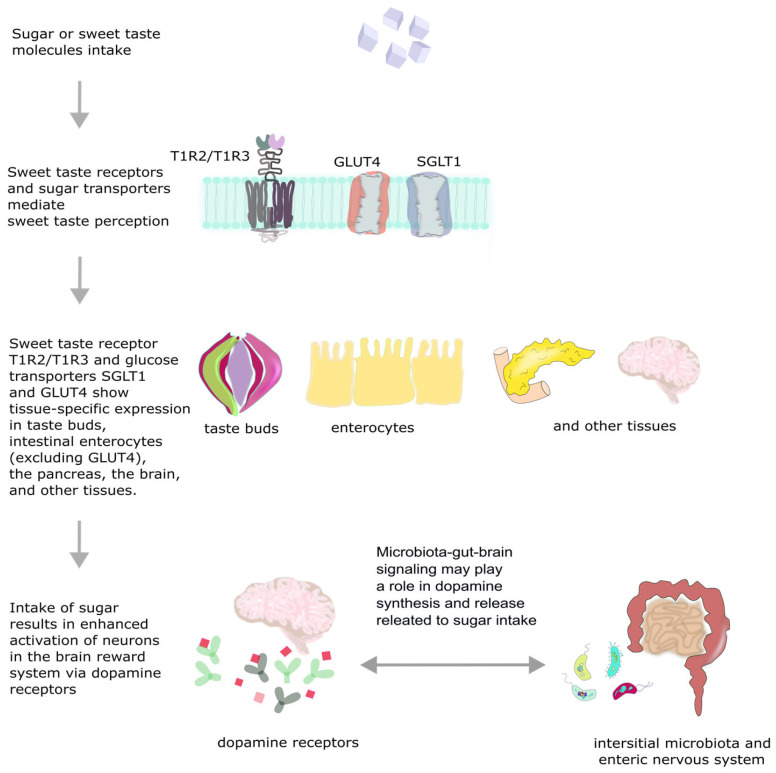
Schematic representation of sugar sensation and signaling in the human body. This figure illustrates pathways through which sugar consumption affects the human body. Sweet taste receptors (T1R2/T1R3), expressed in taste buds, enterocytes, pancreas, brain, and other tissues, transduce signals of sweet-tasting compounds to the brain. Sweet taste perception might also be mediated by SGLT1 and GLUT4 transporters present on the tongue. In the gut, SGLT1 acts as a conduit between the intestine and vagal nerve, taking part in the transduction of sweet taste to the brain. In the brain, sugar activates the dopaminergic system, regulating behavioral responses in relation to reward. Conversely, through afferent pathways, the brain modulates gastrointestinal activity via the microbiota-gut-brain axis that may further modulate dopamine metabolism related to sugar intake. The arrows point in the direction of the action.

The brain’s reward-signalling system consists of structures responsible for processing the sensation of rewards, driving motivation, and contributing to the experience of pleasure [23]. It is composed of various structures, including the VTA, nucleus accumbens (NAc), prefrontal cortex (PFC), hippocampus, and amygdala [20,28]. There are two independent dopaminergic circuits: the mesolimbic system that projects from VTA to the ventral striatum, and the nigrostriatal system that projects from the substantia nigra pars compacta to the dorsal striatum. Oral intake of sugar contributes to enhanced activation of neurons in both circuits [24]. A schematic representation of sugar sensation and signaling in the human body is presented in Figure 1.

Bijoch et al. conducted a study comparing the effects of sucrose and cocaine on neural activity in different parts of the reward system [29]. Fos proto-oncogene, AP-1 transcription factor subunit (C-fos) is a protein of early response expressed in neurons activated by impulses such as those related to reward. C-fos was used as a marker of brain activity to create a neuronal map of silent synapses across various brain regions in response to sugar and cocaine exposure. With the use of magnetic resonance imaging (MRI), a decrease was detected in the activation of mice’s distant brain regions in response to reward. Some regions were activated only by sucrose or cocaine or both of them. In addition, the growth was observed as well in the presence of silent synapses in NAc, serving as an indicator of synaptic reorganization that is observed in cocaine addiction. Both sugar and cocaine exposure resulted in an increase in silent synapses in dopamine receptor D1 and dopamine receptor D2. This observation suggests that sugar may cause similar neuronal changes as addictive substances [29].

In humans, a high-sugar diet is associated with neuro-behavioural impairment. Supporting evidences come from observation that binge eating and drug addiction involve overlapping neural systems, with dopamine, opioid, and cannabinoid systems regulating both feeding behaviors and addiction [7]. Sugar appears to affect the dopamine system in a cocaine-like manner, contributing to decreased basal activity in frontal areas linked to reward regulation and self-control [20,29,30]. Response of the nucleus accumbens is similar to the one observed after consumption of psychostimulants. Finally, palatable food might also induce withdrawal or craving for specific kinds of foods that are generally high in fat, salt, and sugar. This reaction may contribute to the changes observed in drug addiction [7].

Furthermore, investigations conducted by Beilharz et al. and other authors have suggested that the western diet, characterized by excessive levels of simple carbohydrates, affects learning and memory functions. It promotes neuroinflammation, gut dysbiosis, and a reduction in dendritic arbor complexity in the hippocampus, which leads to decreased brain plasticity [31,32,33]. Dysregulation of the brain’s reward signalling system can, apart from contributing to metabolic problems, lead to problems with decision-making and selecting behaviours. This can lead to seeking food, overeating, and weight gain with the assistance of AgRP and POMC neurons in the arcuate nucleus, whose activity is influenced by glucose and that regulates appetite [34,35,36]. In addition, Lavielle et al. have shown that patients with impaired glucose tolerance are more vulnerable to engaging in negative eating behaviours or even to food addiction [37].

A matter that requires clarification is the connection between sweet taste perception, food reward, and binge eating [8]. To achieve this, questionnaires evaluating dietary preferences, food consumption frequency, and tendencies toward binge eating are used; however, they often neglect the underlying mechanisms of the reward system and population variability [38]. Furthermore, the perception of sweet taste does not necessarily correlate with impulsive eating behaviour [38]. Only in a few studies, such as those by Spetter et al. and other authors, have linked brain activity (measured by means of MRI) to sweet taste perception [39].

Even though sugar shares some strong similarities with addictive substances, there are still doubts about whether we can speak about sugar addiction [9]. The response to sugar seems to depend on other factors, such as psychological and environmental influences, meaning that it doesn’t meet all the criteria for typical addiction [9,40]. It might suggest that there is no sugar addiction, but rather a preference for the taste of sugar. These observations redirect attention from sugar as a compound to the sensory and rewarding properties associated with its intake—especially sweetness—as a potential cause of overconsumption. Exploration of this hypothesis requires investigating the neurobiological and perceptual foundation of sweet taste [9]. What is more, understanding habitual sugar consumption may contribute to the physiological systems is crucial, as these patterns are suggested to affect not only behavioral outcomes, but also the metabolic changes that influence cardiovascular health [23].

## 3. Refined Sugars in the Diet and Cardiovascular Health Consequences

**Figure 2:**
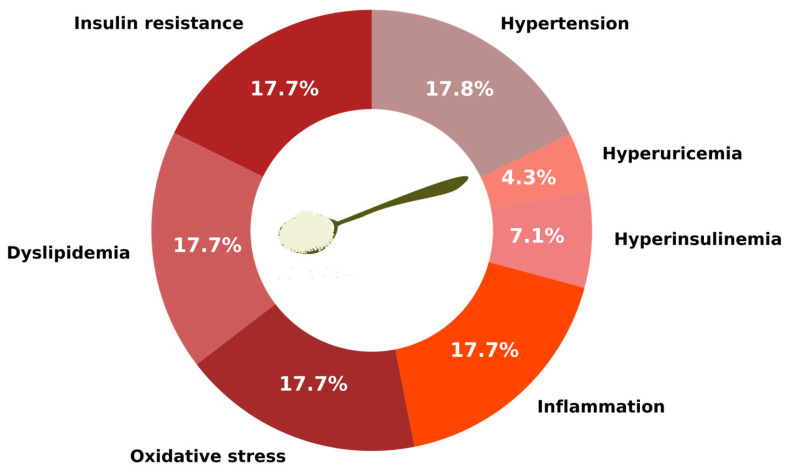
The graph illustrates the proportion of articles published over the past ten years containing the keywords “sugar” and [appropriate term, e.g., hypertension], based on data from the PubMed database. The data were visualized using the Matplotlib package (Python 3.12). The graphical element was drawn manually.

The WHO recommends that the intake of free sugars (including added sugars and those naturally present in honey, syrups, and fruit juices) should be less than 10% of total daily energy intake. Moreover, further reduction to below 5% may provide additional health benefits, especially in the prevention of dental caries and obesity [41]. Excessive consumption of sugars, especially added dietary sugars, plays a significant role in the development of metabolic disorders. These, in turn, can negatively affect cardiovascular health by promoting inflammation, dyslipidemia, and insulin resistance (Figure 2) [5].

### 3.1. Insulin Resistance and Hyperinsulinemia

As SSBs are a major source of glucose, their intake stimulates the secretion of insulin [6,19]. Insulin, in turn, promotes hepatic fatty acid synthesis in hepatocytes, which results in higher synthesis of triglycerides (TG). This process supports the formation and secretion of very low-density lipoproteins cholesterol (VLDL), causing increased concentration of TG in serum [42]. The consumption of SBBs also exerts adverse effects on metabolism by inducing rapid increases in blood glucose and insulin levels. Due to the fact that SSBs typically have a medium or high glycemic index and are often consumed in large quantities, this results in their high dietary glycemic load. This promotes weight gain by elevating the insulin-to-glucagon ratio, which increases hunger and lowers energy expenditure [6].

Notably, there are some differences in the hepatic metabolism of glucose and fructose. After absorption, glucose skips the liver and enters systemic circulation as its hepatic metabolism is regulated by insulin [43]. Fructose, derived generally from HFCS, is almost entirely taken up by the liver, where it is phosphorylated by fructokinase [43,44], an enzyme independent from the metabolic needs of cells, which results in unregulated hepatic fructose metabolism. As a result, fructose overload is an excessive substrate for de novo lipogenesis, increasing lipid accumulation. The accumulation of lipids in the liver may also contribute to the development of hepatic insulin resistance, probably through the level of diacylglycerol (DAG). DAG activates protein kinase C, which results in disrupting insulin signalling. Due to selective insulin resistance, hepatic de novo lipogenesis becomes further upregulated. This creates a vicious cycle in which lipid accumulation worsens insulin resistance, which in turn further stimulates lipogenesis [43].

### 3.2. Dyslipidemia

Fructose contributes to dyslipidemia by increasing the hepatic production and secretion of triglyceride-rich VLDL. Furthermore, it stimulates the synthesis of apolipoprotein CIII (apoCIII), a compound engaged in the development of triglyceridemia. ApoCIII, by participating in the incorporation of lipids into VLDL, promotes the formation of larger, triglyceride-rich VLDL particles. ApoCIII also contributes to increases triglyceride levels by inhibiting their hydrolysis by lipoprotein lipase and by imparing the hepatic clearance of triglyceride-rich VLDL [43]. Elevated levels of triglyceride-rich VLDL and chylomicrons influence the metabolism of other lipoproteins via the action of cholesterol ester transfer protein (CETP). CETP mediates the exchange of triglycerides from triglyceride-rich VLDL and chylomicrons for cholesterol esters from low-density cholesterol (LDL) and high-density lipoproteins cholesterol (HDL). Triglycerides in HDL and LDL are further hydrolyzed by hepatic lipase and lipoproteitn lipase. As a result, LDL and HDL particles become enriched in triglycerides and depleted of cholesterol. This promotes the formation of small, dense LDL and HDL particles-features characteristic of an atherogenic lipoprotein profile [42]. High plasma levels of LDL and VLDL contribute to cardiovascular disease by enhancing atherosclerotic plaque formation and thrombosis. Small, dense LDL particles are particularly susceptible to oxidative modification. VLDL particles further aggravate atherosclerosis by promoting leukocyte migration through interactions with endothelial VLDL receptors and fibrin, facilitating foam cell formation and vascular inflammation. Hypertriglyceridemia affects hepatic metabolism of lipoproteins by reducing their clearance by the liver and increasing plasma triglyceride concentrations [45]. As a result, these lipids and lipoprotein alterations promote the development of atherosclerosis and are associated with an increased risk of coronary artery disease, myocardial infarction, ischemic stroke, and other forms of vascular disease [46].

### 3.3. Oxidative Stress and Inflammation

Sugars present in SSBs and highly processed food are responsible for excessive production of reactive oxygen species (ROS) via different cellular pathways, including mitochondrial dysfunction, activation of specific enzymes, and increased levels of uric acid. Oxidative stress triggers inflammatory reactions—it activates nuclear factor kappa-light-chain-enhancer of activated B cells (NF-κB), increases the expression of adhesive molecules, such as intercellular adhesion molecule 1 (ICAM-1), vascular cell adhesion molecule 1 (VCAM-1), leukocytes, and platelets. Finally, it promotes vascular inflammation and initiates thrombosis. ROS are also involved in atherosclerosis—they oxidize LDL, leading to the formation of oxidized LDL (oxLDL). These are taken up by macrophages, which results in the formation of foam cells responsible for the development of atherosclerotic plaque [19]. Fructose also increases levels of inflammatory factors, such as C-reactive protein (CRP), interleukin-6 (IL-6), tumor necrosis factor receptor 1 (TNFR1), and TNF receptor superfamily. Their presence is responsible for developing atherosclerosis, plaque stability, and thrombosis [47]. Excessive intake of sugars can also lead to increased formation of advanced glycation end-products (AGEs), which play an important role in the regulation of vascular tone. Endothelial dysfunction is a hallmark of several chronic conditions, including hypertension and diabetes. It is also closely associated with impaired nitric oxide (NO) signalling. This dysfunction is not mainly caused by lower production of endothelial nitric oxide synthase (eNOS), but rather by reduced activation of the enzyme or by faster breakdown of NO due to its reaction with ROS. This reduces the elasticity of vessels and promotes hypertension. Another important mechanism involves glycation of mechanosensory proteins responsible for detecting shear stress induced by blood flow. Chronic conditions promote the accumulation of AGEs, which modify proteins, causing loss of their sensitivity. As a result, eNOS activation is impaired, and it disturbs the activation of NO and the function of vessels. Chronic exposure to oxidative stress impairs mitochondrial function in vascular cells, disturbing homeostasis of calcium and initiating apoptosis. As a result, chronic endothelial and microvascular dysfunction, characterized by increased oxidative stress, reduced NO, and activation of NF-κB, promotes elevated vascular resistance and activates the renin-angiotensin-aldosterone system (RAAS). Persistent pressure and oxidative stress stimulate cardiomyocyte hypertrophy and interstitial fibrosis through signalling pathways involving transforming growth factor-β (TGF-β) and angiotensin II. Consequently, vascular and myocardial remodelling occur, which contributes to the development of cardiomyopathy, heart failure, and arrhythmias [19,48].

### 3.4. Hypertension and Hyperuricemia

Hypertension serves as the most common preventable risk factor for CVD, including coronary heart disease, heart failure, stroke, myocardial infarction, atrial fibrillation, and peripheral artery disease [48]. Xi et al. in their meta-analysis demonstrated that higher consumption of SSB was associated with an increased risk of hypertension and coronary heart disease. The underlying mechanism is largely attributed to the elevated fructose intake [47]. Fructose is rapidly phosphorylated in hepatocytes, leading to AMP accumulation, activation of AMP deaminase, and increased uric acid production [49]. Fructose contributes to both acute and chronic elevations of serum uric acid, which affects NO production in the endothelium, causing decreased vasodilation, leading to increased vascular resistance and elevated blood pressure. Moreover, fructose activates the RAAS, resulting in increased blood volume, sodium retention, and renal microvascular damage, all of which contribute to the development and persistence of hypertension [42,47]. What is more, elevated uric acid levels induced by fructose metabolism may contribute to oxidative stress and inflammation, causing endothelial dysfunction. Uric acid stimulates the production of ROS and promotes vascular proliferation, both of which play crucial roles in vascular remodelling. These processes not only aggravate hypertension but also accelerate the development of atherosclerosis and left ventricular hypertrophy [49,50]. Additionally, sugar intake also stimulates activation of the sympathetic nervous system, leading to elevated heart rate and vascular resistance [42].

### 3.5. Sweet Taste and Obesity

Even though mechanisms of sweet taste perception are well established, current evidence on the association between this pathway and obesity is insufficient [8]. Ribeiro et al. have conducted research in which they compared sweet taste perception in 247 individuals with severe obesity and 174 healthy volunteers, suggesting that the first group was more sensitive to sweet taste than the second one [51]. Sartor et al. have reached an alternative conclusion in this area [52], while Pepino et al. showed that there were no significant changes in taste sensitivity according to patients’ body mass index (BMI) [53]. Discrepancies in this area might be a result of a problem with measuring taste perception—in light of its complexity, sweet taste sensation is a result of intensity, recognizability, pleasantness, or detection threshold that are considered equivalent, but in fact they refer to different sensory and affective mechanisms [51].

Except for discrepancies, the meta-analysis of Trius-Soler et al. reveals some important arguments that higher BMI relates to a higher detection threshold for sweet taste. At the same time, a reduced sensitivity to the stimulus. This study suggests a role for inflammatory factors in the modulation of taste perception [54]. Additionally, other studies point out that this process is reversible. According to studies of Kotackova et al. and Alshwaiyat et al., invasive as well as non-invasive interventions may reduce body weight, while simultaneously lowering the levels of pro-inflammatory cytokines such as CRP. These changes, in turn, can affect the functioning of taste buds and taste neurons [17,55,56].

Another interesting aspect is how sugar intake may influence satiety. Analyses conducted on *Drosophila melanogaster* sugar may disrupt the mechanisms responsible for regulating satiety. The authors demonstrated that high sugar intake may contribute to the function of neurons responsible for the processing of sweet taste. This process can weaken the sensation of satiety and lead to higher sugar intake. Although it has been studied in the fruit fly, there is evidence suggesting that a similar mechanism might potentially occur in humans [57].

Sweet taste perception has also been shown to be modulated by intestinal microbiota [20]. There is growing evidence supporting a correlation between different dietary patterns and the composition and function of microbiota. At the same time, microbiota is suggested to influence the effects of dietary exposures through microbe-host interactions. The mechanism underlying these interactions occurs via various pathways. One of these involves the secretion of neurohormones and neurotransmitters such as GLP-1, peptide YY (PYY), ghrelin, or serotonin; dysbiosis may contribute to dysfunction in their production. This disrupts the proper perception of hunger and satiety. Moreover, lipopolysaccharides (LPS), which are produced in dysbiosis and the associated increase in intestinal permeability, interfere with neural reward and appetite pathways. Through the vagal nerve and inflammation, LPS activation raises dopaminergic activity related to reward, potentially increasing the motivation to consume sugar [58].

These relations have been demonstrated in various studies. Schwarz et al., during their study, have shown that microbiota might modulate the function of sweet taste and glucose transport receptors in the gut, which affect feeding behaviour. Dysbiosis can create increased sensitivity to sugar and its consumption. They observed increased expression of T1R1 and SGLT-1 receptors in enterocytes of obese, germ-free mice—these mice consumed more sucrose compared to mice with normal microbiota [59]. The crucial role of microbiota has also been shown in the study by Bernard et al., who investigated the influence of prebiotic supplementation (10% inulin-type fructans) in obese mice. This intervention not only corrected dysbiosis but also increased sensitivity to sweet stimuli with a better hedonic response without motivational change. This resulted in a correction of the preference [60]. Miras et al. have shown that after gastric bypass surgery, which contributes to altering the gut microbiota, some changes in taste receptor expression and their activity occurred, affecting patient satiety and food preferences [61].

Finally, sweet taste perception and consumption are regulated not only by sensory signals but also by cognitive-social factors [10]. Attitudes toward sugar, nutritional knowledge, self-efficacy in dietary control, and general health beliefs significantly influence the consumption of sugar and sweetened products [11,12].

This highlights the importance of searching for sugar alternatives that satisfy the desire for sweetness while also providing nutritional and bioactive benefits, without negatively affecting cardiometabolic health [62].

## 4. Fruits as a Remedy for Cardiovascular Health

Studies show that sugar intake is part of a broader pattern of eating behaviours and should be analysed in the context of psychosocial factors and lifestyle. Consequently, reduction of sugar intake requires educational and behavioural changes [11,12].

In this context, naturally occurring sweet compounds are derived from plants, attracting attention as potential sugar alternatives [13]. These compounds are interesting not only due to their natural sweetness and favorable sensory properties, but especially regarding cardiovascular health [63]. Considering fruit as a great source of such bioactive compounds, it may offer natural health-promoting benefits to reduce sugar intake [13].

### 4.1. Plants as a Natural Source of Sweetness

The taste of fruits is attributed to the presence of carbohydrate compounds as sucrose, fructose, glucose [64]. In addition to these sugars, some plants contain sugar alcohols (polyols), such as sorbitol, mannitol, maltitol, and xylitol [65]. Moreover, many types of fruits, particularly those of tropical origin, also contain protein capable of activating taste receptors [66,67]. Even though present only in trace amounts, these compounds can be up to several thousand times sweeter than sucrose. That makes them of particular interest not only from the perspective of taste physiology but also as potential substitutes for table sugar [68]. There is also another group of plant-based sweeteners like glycosides, that can bind to sweet taste receptors. These substances may be natural, non-nutritive alternatives to sugar or may modulate sweet taste perception, such as gymnemic acids, the triterpenoid glycosides [69]. Mogroside V is a sweet-tasting representative of glycosides found in fruits.

**Figure 3:**
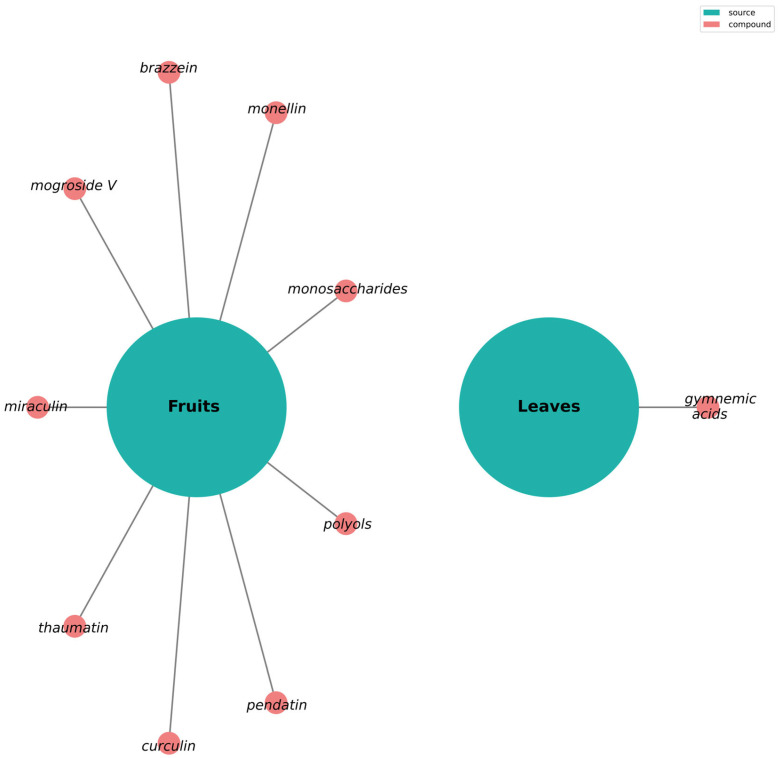
The graph illustrates a source of sweet taste in fruits and sweet taste-modulated compounds in leaves. The large green node is arranged at the center, while associated sweet compounds, including monosaccharides, polyols, and sweet protein nodes, are positioned radially (depicted as small dark red nodes). Gymnemic acids are shown as a small dark red node linked with the second large green node, because of their ability to modulate sweet taste receptors without possessing a sweet taste and having no fruit origin. Data were visualized using the NetworkX package in Python 3.12.

Meta-analyses show that natural, plant-based sweeteners are suggested to have a generally healthier profile than synthetic sweeteners, particularly regarding gut microbiota and glucose metabolism [70]. Figure 3 illustrates sweet taste and sweet-taste modulated compounds in plants.

#### 4.1.1. Sweet Proteins

Monellin is a naturally occurring sweet protein found in *Dioscoreophyllum cumminsii*, a plant classified with the class *Magnoliopsida*, order *Ranunculales*, and family *Menispermaceae*, which is native to West Tropical Africa [66,71]. In several African countries, it has traditionally been used both as a raw edible fruit and as a natural sweetener for food products [71]. Structurally, monellin is a polypeptide composed of 94 amino acids (aa), forming two separate chains, with a molecular mass of approximately 11 kilodaltons (kDa). It is 2000 times sweeter than sucrose [66]. This compound has several metabolic advantages—it has low-calorie content and low glycaemic index, making it a potentially dietary component in a diabetic diet [72]. Furthermore, Cancelliere et al. have shown the effects of an isolated monellin-derived aa chain (MNEI) in obese rodents fed a high-fat diet. The intake of fluids supplemented with MNEI resulted in improved insulin sensitivity and favourable alterations in lipid profiles in the tested animals [73]. Sensory analysis shows that monellin provides a clean, high-quality sweetness with no lingering bitterness [62].

Brazzein is found naturally in the fruits of *Pentadiplandra brazzeana*, a plant native to the tropical forests of Africa. In this plant, brazzein exists in two naturally occurring forms: the major form, which accounts for approximately 80% of the protein content and is predominantly present in the seeds, and the minor form, comprising about 20%, which is reported to be even sweeter [72]. In African regions, brazzein is traditionally used as a natural sweetener for food and beverages [66]. This substance is the smallest known sweet protein, consisting of a single polypeptide chain of 54 aa, yet it is approximately 750 times sweeter than sucrose on a weight basis [66,72]. In their study, Chung et al. have suggested that brazzein exhibits antioxidant, anti-inflammatory, and anti-allergic properties [74].

Miraculine is a sweet-tasting glycoprotein found naturally in the fruits of *Richardella dulcifera*, a plant native to the western parts of Africa. It is composed of 191 aa and functions as a tetramer. Although miraculin does not elicit a sweet taste on its own, it has the unique ability to make sour flavours, making them taste sweet [75]. The anti-inflammatory potential of miraculin was suggested in a study by Álvarez-Mercado et al., which investigated its effects in oncological patients suffering from dysgeusia and malnutrition. Participants were given tablets containing lyophilized *Synsepalum dulcificum* fruit, rich in miraculin, in varying doses prior to each main meal. The intervention led to a reduction in inflammatory cytokines, including IL-6, Interleukin 1 β (IL-1β), and TNF-α [76].

Thaumatin is a naturally present protein in the fruits of the tropical *Thaumatinococcus danielli*. It’s 3000 times sweeter than sucrose. Research proved that it’s not allergic or toxic. Due to that fact, it has already been allowed to serve as a flavour modifier and intense sweetener in different countries [75]. Thaumatin, whose sweetness develops slowly, at sub-sweetening concentrations it enhances specific flavors such as mint and coffee [62]. Its consumption is considered safe, as it does not cause tooth deterioration and is suitable for individuals with diabetes.

Curculin is obtained from the fruit of *Curculigo latifolia*, a plant native to certain regions of Malaysia. It is built of 114 aa and consists of two identical polypeptide chains. Curculin is approximately 550 times sweeter than sucrose. It functions as both a flavor enhancer and a taste-modifying protein capable of converting sour flavors into sweet [75]. Curculin is heat-sensitive, and its sweet taste properties reappears if the subject is drinking water [62].

Pentadin naturally occurs in the fruits of *Pentadiplandra brazzeana*, a plant native to Africa, particularly found in Gabon. It is a protein with a molecular weight of approximately 12 kDa and is about 800 times sweeter than sucrose [75].

#### 4.1.2. Glycosides and Sweetness Perception

Mogroside V is a naturally occurring sweet glycoside of *Siraitia grosvenorii*, commonly known also as monk fruit, that belongs to the family of Cucurbitaceae. Native to the southern parts of China, it has been cultivated in specific regions for over 200 years. The fruit, characterized by sweet and fleshy pulp, has been widely used in traditional Chinese medicine for the treatment of respiratory tract conditions, such as lung congestion, colds, and sore throats. The sweetness of monk fruit is attributed to cucurbitane-type triterpene glycosides—mogrosides [77]. Mogroside V is approximately 200 to 300 sweeter than sucrose [23]. Its advantages include low caloric content and a range of potential health-promoting properties, such as anti-cancer, anti-diabetic, anti-inflammatory, and antioxidant activities [77]. The effect of mogroside V on microbiota has been analysed in the study of Xiao et al. In this study, several beneficial effects were shown, including modulation of beneficial gut microbiota. At the same time, a reduction in the presence of potentially pathogenic bacteria and enhanced production of key short-chain fatty acids (SCFAs) were associated with the intake of mogroside V [78]. Antidiabetic function was shown by Qin et al. in their study, mogroside treatment significantly improved hepatic glucose metabolism in type 2 diabetic rodents, reduced plasma endotoxin and inflammatory factor levels. What is more, it reduced the level of *Firmicutes* and *Proteobacteria* and increased the level of *Bacteroidetes*, which indicates that mogroside plays a therapeutic role as an intestinal microbiota regulator in the treatment of type 2 diabetes mellitus [79]. Finally, Wang et al. have suggested the role of mogroside in modulating microbiota in obese mice. It reduced body weight gain and fat tissue weight of the mice fed with a high-fat diet, and improved glucose tolerance and insulin sensitivity. According to microbiota profiling in this study, the abundance of *Firmicutes* was increased and the abundance of *Bacteroidetes* was decreased, resulting in a higher *Firmicutes* to *Bacteroidetes* (F/B) ratio. That ratio was restored to the control levels by treatment of mogroside [80].

Based on both studies, it can be observed that supplementation with mogroside V has a beneficial effect on restoring a healthy microbiota, as evidenced by a decrease in *Firmicutes* and an increase in *Bacteroidetes* [79,80].

Although intensive research continues on substances that can replace naturally occurring sugars—such as sweet proteins or artificial sweeteners—natural compounds capable of modulating the perception of sweet taste are gaining increasing scientific interest [21,75]. These substances do not possess inherent sweetness but interact with sweet taste receptors at the molecular level. That effect leads to a reduction in or temporary suppression of sweet taste perception. Such an outcome may be particularly relevant in populations at risk of excessive sugar consumption and its associated metabolic consequences. One of the best-characterized compounds of this type is gymnemic acid - glycoside found in the leaves of the plant *Gymnema* [81].

*Gymnema* is a genus belonging to the *Apocynaceae* family, comprising more than 50 species of *Gymnema* around the world [82]. Among them, *Gymnema sylvestre*—the plant native to central and western India, tropical Africa, and Australia—is one of the best-known species. This plant is popular as a rich source of numerous bioactive compounds that are widely used in traditional medicine in the treatment of diabetes, malaria, asthma, eye complaints, inflammations, and snakebite. Moreover, it exhibits antihypercholesterolemic, hepatoprotective, and sweet taste-suppressing properties [83,84]. Other notable species within the genus include *Gymnema lactiferum*, the plant that has properties similar to those of *Gymnema sylvestre* [85]. *Gymnema inodorum* is another herbal species containing gymnemic acids, which are primarily responsible for the suppression of sweet taste perception [82,83,85].

Gymnemic acids are an oleanane-type triterpene glycoside that may appear as an individual compound or as a complex of structurally related compounds. The major saponin fraction in *Gymnema sylvestre* is a gymnemic acid that comprises a heterogeneity of at least nine molecules [86]. Among them, the main ones, gymnemic acid A–D, are found in every part of the plant, with the highest concentration in shoot tips and the least in seeds [83]. The mechanism of action of gymnemic acids is multifaceted. As a taste modulator, they selectively and temporarily inhibit sweet taste without affecting other tastes like salty, sour, bitter, and umami. They bind to taste receptor type specifically TAS1R2 and TAS1R3 on the tongue and palate, preventing the interaction of sugar molecules with these receptors. Consequently, the activation of the chorda tympani nerve, responsible for transmitting sweet taste signals to the brain, is temporarily inhibited. The sweet taste inhibition generally lasts 30 to 60 min [87].

In terms of glycaemic control, gymnemic acids exert multiple antihyperglycemic effects: increasing insulin, promoting regeneration of pancreatic islet cells, and increasing the peripheral utilization of glucose. This involves upregulation of glucose-metabolizing enzymes involved in insulin-dependent pathways, including increased phosphorylase activity and downregulation of gluconeogenic enzymes and sorbitol dehydrogenase [84]. In the intestine, gymnemic acid binds to the receptor present in the superficial layer of the intestine, inhibiting the absorption of glucose and contributing to a reduction in postprandial blood glucose levels. Gymnemic acid has been known to interact with glyceraldehyde-3-phosphate dehydrogenase (GAPDH), taking part in the antihyperglycemic properties [83].

Several studies have demonstrated that gymnemic acids influence sweet taste perception and hedonic responses to high-sugar foods. A study by Turner et al. has suggested that gymnenic acids reduced both the pleasantness and the desire to consume high-sugar foods, particularly in individuals who self-identified as having a “sweet tooth”. Participants also reported lower intake of sweet foods compared to placebo controls, suggesting that gymnemic acids can reduce hedonic appeal rather than affect hunger or energy needs directly [81]. Stice et al. have shown that an acute dose of *Gymnema sylvestre* lowered reward region activation in the brain in response to both cues associated with sweet beverages and the actual taste of those beverages. This study suggests that *Gymnema sylvestre* can contribute to long-term reduction of sugar intake [88].

Beyond its effects on sweet taste and glucose metabolism, *Gymnema inodorum* has demonstrated significant antioxidant potential. Saiki et al. purified gymnemic acids from the plant and have suggested that by inhibiting 3T3-L1 preadipocyte cell line differentiation into adipocytes and suppressing genes that are associated with adipogenesis, they may contribute to the prevention of obesity [86].

In addition, *Gymnema sylvestre* hydroalcoholic extract (HAEGS) has been shown to exert protective effects against acute respiratory distress syndrome induced by LPS. Jangam et al. have shown that the extract, particularly fraction 6 enriched in gymnemic acids, demonstrated anti-inflammatory and antioxidant activities by inhibiting key inflammatory signalling cascades [89].

In the study of Rachh et al., rats administered a high-cholesterol diet exhibited elevated serum levels of total cholesterol (TC), triglycerides, LDL, and VLDL, accompanied by a reduction in HDL. Subsequently, these animals were treated with an HAEGS of *Gymnema sylvestre* leaves. Intervention with HAEGS of *Gymnema sylvestre* leaves resulted in significantly improved lipid profiles, reducing harmful lipid fractions and increasing HDL levels. These results suggest that gymnemic acids may support managing hyperlipidaemia and associated metabolic disorders [90].

### 4.2. Fruits as a Source of Variety of Bioactive Compounds

Fruits are rich in numerous bioactive compounds that contribute to health benefits, including the prevention of chronic diseases [91,92]. Since such bioactive compounds occur in fruits, vegetables, and other edible plant parts, they are frequently discussed jointly in the literature as common sources of protective agents [83,85,92].

#### 4.2.1. Flavonoids

Flavonoids are polyphenolic compounds naturally found in fruits, vegetables, cereals, and plant-based beverages. Flavonoids have a fifteen-carbon flavone skeleton consisting of two benzene rings (A and B) connected by a three-carbon heterocyclic pyran ring (C) [93]. Based on their structural features, flavonoids are classified into twelve main subclasses. Six of them have dietary importance and include anthocyanidins (e.g., pelargonidin, cyanidin), flavan-3-ols (e.g., epicatechin, epigallocatechin), flavonols (e.g., quercetin, kaempferol), flavones (e.g., luteolin, baicalein), flavanones (e.g., hesperetin, naringenin), and isoflavones (daidzein, genistein) [94]. The health benefits of flavonoids stem from their structural characteristics, such as the number and position of hydroxyl groups and the presence of C2C3 double bonds. This determines their ability to chelate metal ions, terminate reactive oxygen species, and interact with biological factors to induce a biological response [95]. Flavonoids have powerful treatment potential for cardiovascular diseases. They play a primary role in vasodilation, platelet function, free radical scavenging, lipoprotein regulation, antioxidant properties, action on blood vessel walls, mitochondrial protection, and improved cardiomyocyte fibrosis [96]. Cohort studies and meta-analyses have shown that regular consumption of flavonoids is associated with the prevention of cardiovascular diseases, especially atherosclerosis [95]. In a cohort study involving 49,173 participants, an inverse association was observed between dietary intake of flavonoids, flavanones, dihydrochalcones, and isoflavonoids and the risk of all-cause mortality [97]. Similar findings were observed in a 23-year cohort study of 56,048 participants, which demonstrated that regular flavonoid consumption was inversely associated with cardiovascular and cancer mortality [98]. A systematic review of cohort studies found that the intake of anthocyanidins, proanthocyanidins, flavones, flavanones, flavonols, and flavan-3-ols was inversely associated with the risk of cardiovascular disease. A total of fourteen prospective cohort studies were included in the study. This work indicates that dietary intake of six classes of flavonoids has a significant impact on reducing the risk of cardiovascular disease [99]. Furthermore, a meta-analysis of 20 studies involving 856 healthy individuals demonstrated that cocoa products (cocoa or chocolate) rich in flavanols (average intake of 545.5 mg) lowered blood pressure in short-term studies (2–18 weeks). In summary, a diet rich in flavanols can significantly lower blood pressure by an average of 2–3 mm of mercury (mm Hg), with the cacao fruit itself—also edible—providing small additional amounts of flavonoids [100].

#### 4.2.2. Phenolic Acids

Phenolic acids are typically divided into hydroxycinnamic acids and hydroxybenzoic acids. These compounds have a phenyl group substituted with one carboxyl group and at least one hydroxyl (OH) group [101]. Hydroxybenzoic acids include salicylic acid, protocatechuic acid, vanillic acid, and gallic acid. Examples of hydroxycinnamic acids include p-coumaric acid, caffeic acid, and chlorogenic acid [102]. They are found in various fruits and vegetables, cereals, spices, coffee, and tea, and participate in numerous biochemical processes. Phenolic acids are insoluble and, for the most part, occur in combination with other molecules or components [103]. The most important health-promoting properties of phenolic acids stem from their antioxidant activity. They are antioxidants, compounds that have the ability to prevent the production of reactive oxygen species. This stems from their chemical structure, specifically the presence of a hydroxyl substituent on the aromatic ring. Several mechanisms of their action are known. The main one is the scavenging of free radicals by donating hydrogen atoms. Substituents on the aromatic ring influence the stability of the structure and, consequently, the ability to scavenge free radicals [102]. Phenolic acids have a wide range of effects on the body, some of which include anti-inflammatory, anticancer, antimicrobial, antioxidant, hepatoprotective, cardioprotective, immunomodulatory, neuroprotective, and metabolic-regulating effects [104,105]. The anti-inflammatory activity of phenolic acids is related to their effects on immune cells. They can both inhibit and modulate the expression of proinflammatory cytokines and chemokines, including TNF-α, IL-1β, IL-6, and IL-8 [106]. Thanks to their antioxidant properties, phenolic acids reduce the oxidation of LDL lipoproteins and platelet aggregation. This action is responsible for, among other things, preventing cardiovascular diseases. Furthermore, they prevent the development of vascular inflammation and endothelial dysfunction, which play a significant role in the development of these diseases [107,108]. Phenolic acids affect the function of insulin and glucose receptors. They also inhibit two key enzymes: α-glucosidase and α-amylase. These enzymes are responsible for converting dietary carbohydrates into glucose. Some acids, such as ferulic acid and chlorogenic acid, increase the expression of the glucose transporter GLUT2 in pancreatic β-cells. They also promote GLUT4 translocation via the phosphoinositide 3-kinase/protein kinase B (PI3K/Akt) and AMP-activated protein kinase (AMPK) pathways. As a result, they act as antidiabetic agents [109].

#### 4.2.3. Anthocyanins

Anthocyanins are glycosides of anthocyanidins, flavonoid derivatives produced in the phenylpropanoid pathway. Anthocyanins belong to the class of polyphenols, water-soluble pigments found primarily in fruits and flowers. These compounds are responsible for the red, purple, and blue pigmentation of flowers, seeds, and fruits [110]. The best sources of anthocyanins are berries such as strawberries, blueberries, blackberries, blackcurrants, redcurrants, and raspberries. Elderberries and chokeberries contain the highest amounts of anthocyanins. Other sources of anthocyanins include cherries, plums, pomegranates, eggplants, grapes, and red/purple vegetables such as black carrots, red cabbage, and purple cauliflower [111]. Anthocyanin pigments are used as natural food colorings. However, the color and stability of these pigments are influenced by many factors, including potential of hydrogen (pH), light, temperature, and structure. In an acidic environment, anthocyanins exhibit a red color, while in an alkaline pH, they turn blue [112]. In addition to their use as natural colorants, anthocyanins exhibit a number of health-promoting properties. Numerous studies have demonstrated their antioxidant, antimicrobial, neuroprotective, and anticancer effects, as well as their ability to prevent the onset and progression of numerous diseases affecting the cardiovascular, endocrine, digestive, urinary, and immune systems [111,112,113,114]. Research indicates that anthocyanins may potentially contribute to the prevention and treatment of cardiovascular diseases by improving lipid profiles and vascular function, reducing blood glucose levels and blood pressure, and reducing inflammation [112,115,116]. At the cellular and molecular levels, anthocyanins and their metabolites may protect endothelial cells against aging, apoptosis, and inflammation [115]. Moreover, these compounds limit the proliferation and migration of vascular smooth muscle cells induced by platelet-derived growth factor (PDGF) or TNF-α by inhibiting focal adhesion kinase (FK) and extracellular regulated protein kinase signalling pathways [116]. Anthocyanins may contribute to ameliorating vascular inflammation by reducing the formation of oxidized lipids, preventing the adhesion and infiltration of the vessel wall by leukocytes, and phagocytosis of deposited lipids by macrophages [117]. Conversely, these compounds may reduce the risk of thrombosis by inhibiting platelet activation and aggregation through lowering the levels of P-selectin, transforming growth factor 1 (TGF-1), and CD40 ligand [112,115].

#### 4.2.4. Carotenoids

Carotenoids are a group of fat-soluble red, yellow, and orange organic pigments found in many foods, such as fruits and vegetables. Based on their molecular composition, carotenoids are divided into carotenes and xanthophylls [118]. Carotenes are hydrocarbon molecules composed solely of hydrogen and carbon atoms, without functional groups. This group includes lycopene, α-carotene, and β-carotene. Xanthophylls, on the other hand, are hydrocarbons that also contain oxygen, and examples include β-cryptoxanthin, lutein, zeaxanthin, violaxanthin, neoxanthin, fucoxanthin, astaxanthin, capsanthin, bixin, and crocin [119]. The main food sources of these compounds include: α-carotene: carrots, winter squash, and butternut squash; β-carotene: dark orange and green fruits and vegetables; Lycopene: tomatoes and tomato products; β-cryptoxanthin: tropical fruits and sweet red peppers; lutein: leafy vegetables, corn, and green vegetables; zeaxanthin: egg yolk, corn, cornmeal, and leafy vegetables [118]. β-carotene, α-carotene, γ-carotene, and β-cryptoxanthin act as precursors of vitamin A. It is known that all carotenoids have strong antioxidant properties, neutralizing intracellular ROS [120]. Antioxidant compounds reduce the oxidation of LDL in cardiovascular tissues, contributing to slowing the atherosclerotic process. In addition to their antioxidant effects, carotenoids also positively affect endothelial function, lower inflammatory markers, and improve lipid profiles, reducing a number of risk factors for cardiovascular disease through various molecular mechanisms [121,122,123]. Lycopene has anti-inflammatory properties and helps inhibit the synthesis of IL-1, IL-6, and TNF-α. It also positively affects lipid profiles by reducing TG, inhibiting LDL oxidation, and improving endothelial function while maintaining NO levels [121]. Lutein inhibits NF-kB transcription and reduces inflammation-related molecules such as TNF-α, IL-6, and prostaglandin E2 (PGE-2). It also helps control systolic blood pressure [119]. Zeaxanthin influences the response to oxidative stress by reducing oxidized glutathione and increasing intracellular amounts of reduced glutathione. glutathione. Higher levels of zeaxanthin have been associated with reduced carotid intima-media thickness [124]. Carotenoids, including β-carotene, exhibit antioxidant and anti-inflammatory effects: they reduce LDL oxidation, modulate nitric oxide bioavailability, inhibit NF-κB activation, and decrease the production of proinflammatory cytokines [120,122]. Through these biological activities, carotenoids positively influence lipid metabolism and endothelial function, suggesting potential benefits for cardiovascular disease prevention and therapy.

#### 4.2.5. Phytosterols

Phytosterols, also known as plant sterols, are components of plant cell membranes and are found in vegetable oils such as corn oil, soybean oil, and rapeseed oil, as well as in grains, nuts, fruits, and vegetables. They are chemical analogues of cholesterol, an animal sterol. They include saturated compounds-stanols-and unsaturated compounds-terols. The most common phytosterols in the human diet are β-sitosterol, campesterol, and stigmasterol [125]. Phytosterols exhibit a range of potential therapeutic effects, including lipid regulation, antioxidant and anti-inflammatory effects, and stabilization of atherosclerotic plaques and vascular inflammation. These substances may represent non-pharmacological treatments for hyperlipidemia when incorporated into functional foods [126]. The protective effect of phytosterols on the cardiovascular system is based on lowering serum LDL levels by reducing the intestinal absorption of dietary cholesterol [127]. These substances compete with cholesterol for incorporation into micelles in the gastrointestinal tract, resulting in reduced cholesterol absorption. This leads to reduced cholesterol delivery to the liver and increased expression of LDL receptors, which lowers LDL levels [125]. Plant sterols or stanols at a dose of 3 g daily lower LDL levels by approximately 12%. Higher doses do not significantly further reduce LDL levels, and lower doses have less impact on LDL. Fruits contain only small amounts of phytosterols; therefore, achieving higher doses requires the consumption of foods enriched with phytosterols, such as margarine or yogurt [42,126,128].

#### 4.2.6. Dietary Fiber

Dietary fiber is an indigestible carbohydrate, including non-starch polysaccharides, cellulose, pectins, hydrocolloids, fructooligosaccharides, and lignin. It is found primarily in fruits, vegetables, whole grains, nuts, seeds, and legumes. It can be divided into two main types: soluble and insoluble. The main sources of soluble fiber are fruits and vegetables, while insoluble fiber comes from cereals and whole grains [129]. Dietary fiber has a wide range of health benefits: it regulates intestinal function, prevents constipation, helps control blood glucose and cholesterol levels, increases satiety, and positively affects the intestinal microbiota, which is important for immunity and overall health. Regular fiber consumption is also important in preventing cardiovascular disease, type 2 diabetes, and certain cancers, especially colon cancer [129,130,131,132,133]. Studies have shown that higher intake of total dietary fiber—both soluble and insoluble—is associated with a reduced risk of cardiovascular events [42,132]. Studies have shown that increased dietary fiber lowers TC, LDL, and favorably alters HDL levels. Dietary fiber is suspected of reducing cholesterol absorption by the small intestine. This contributes to a reduction in cholesterol content in chylomicrons and reduced cholesterol delivery to the liver. Reducing cholesterol in the liver increases the expression of LDL receptors, which leads to a reduction in plasma LDL concentrations. Dietary fiber may also reduce the absorption of bile acids in the small intestine, resulting in increased utilization of hepatic cholesterol for bile acid synthesis. This effect also reduces hepatic cholesterol by increasing LDL receptor expression, thus lowering plasma LDL concentrations. Furthermore, it has been concluded that fermentation of dietary fiber in the large intestine is associated with the production of short-chain fatty acids, such as acetate, propionate, and butyrate, which inhibit cholesterol synthesis in the liver, contributing to a reduction in LDL concentrations [42,132,133,134].

#### 4.2.7. Bioactive Peptides

Fruits are a valuable source of exogenous bioactive peptides, active molecules with a broad range of health benefits. They are small compounds, typically consisting of 3 to 20 aa linked by covalent bonds, with a molecular mass of less than 6 kDa. These peptide chains predominantly contain lysine, arginine, and proline, along with additional hydrophobic parts [135].

Bioactive peptides exert their effects by interacting with various molecules in the body, most often by inhibiting their activity. Peptides exhibit various health properties, including antimicrobial, antihypertensive, antioxidant, blood-lipid-lowering, opioid, anti-obesity, mineral-binding, antidiabetic, and antiaging effects [136]. Bioactive peptides may help modulate pain perception by binding to opioid receptors in the nervous system, mimicking the effects of endogenous opioids like endorphins. Moreover, certain peptides can bind essential minerals like calcium, iron, and zinc, enhancing their bioavailability and absorption in the human body [137]. Their biological function depends on the aa composition, sequence, and the hydrophobic or hydrophilic nature of the peptides. Initially, these compounds are inactive and embedded within larger protein molecules. They become active through proteolysis, which may occur during enzymatic hydrolysis in the gastrointestinal system, microbial hydrolysis, or chemical hydrolysis.

Bioactive peptides demonstrate promising properties that support cardiovascular health [138]. Angiotensin-converting enzyme (ACE) inhibitory peptides found in peaches and plums are short compounds that help prevent hypertension by blocking the formation of angiotensin II, a vasoconstrictive enzyme that increases blood pressure via its action on blood vessels, the sympathetic nervous system, and adrenal glands. In cherries and coconuts, antioxidant peptides help protect against atherosclerosis by inhibiting the peroxidation of lipids and essential fatty acids. Peptides in sugar apples exhibit anti-inflammatory activity by shielding blood vessels from proinflammatory cytokines, a key factor in atherosclerosis development [139]. Olive-derived peptides contribute by inhibiting endogenous cholesterol synthesis through suppression of the 3-hydroxy-3-methylglutaryl-coenzyme A (HMG-CoA) reductase enzyme [140]. Together with other active compounds like polyphenols and antioxidants, these peptides offer a natural multifaceted defence against cardiovascular disease.

**Figure 4:**
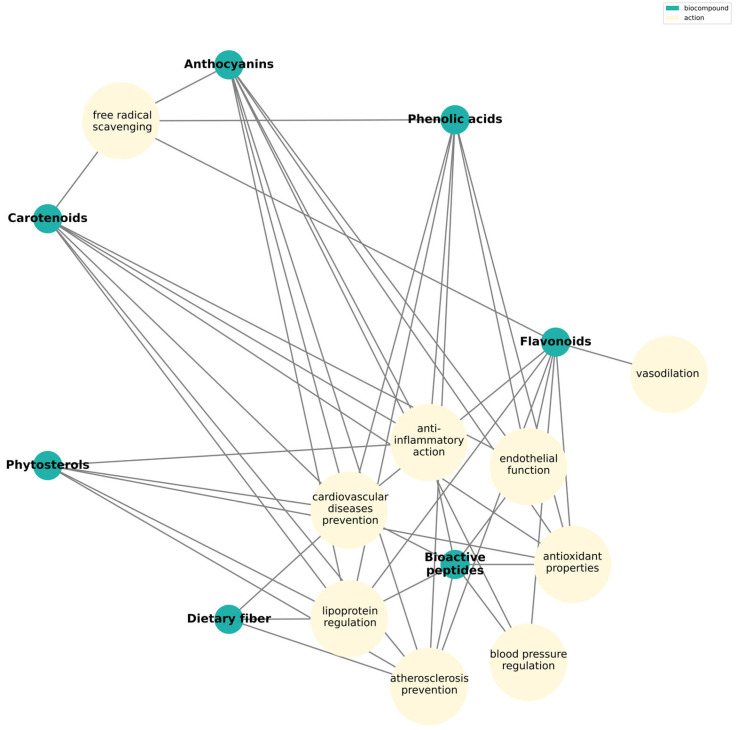
Radial network visualization of bioactive compounds derived from fruits and selected associated clinical phenotypes. The network graph illustrates the relationships between bioactive compounds (smaller green nodes) and their associated clinical processes (large yellow nodes). Bioactive compound nodes are arranged on the outer circle, while associated biological process nodes are positioned radially around each bioactive compound to visualize direct connections. Edges between bioactive compounds and processes represent observed associations. Data were visualized using the NetworkX Python package 3.12.

The bioactive compounds derived from fruits and selected associated clinical phenotypes are summarized in Figure 4. These bioactive compounds contribute to the beneficial effects of fruit-based diets, which have been associated with reduced risk of cardiovascular disease [141].

## 5. Fruit-Based Diets in Cardiovascular Prevention

Collectively, epidemiological, experimental, and clinical data strongly support that fruit-rich diets delay the onset and attenuate the severity of cardiovascular diseases, underscoring the synergistic interplay of their diverse nutrients and phytochemicals [92,141]. Du et al. demonstrated in their study with a cohort of 512,891 Chinese adults that daily fresh fruit consumption significantly reduces the risk of cardiovascular death, major coronary events, ischemic stroke, and hemorrhagic stroke compared to individuals who rarely or never consume fresh fruit [142]. Similarly, higher fruit consumption has been associated with a lower risk of hypertension and decreased carotid intima-media thickness, reflecting reduced atherosclerotic burden [143].

### 5.1. The Role of Specific Fruits in the Prevention of Cardiovascular Diseases

Among fruits with particularly well-documented cardioprotective effects, apples have received substantial attention. Their beneficial impact is largely attributed to high levels of flavonoids and dietary fiber. Research from intervention studies substantiates their positive impact on vascular health. For instance, Bondonno et al. demonstrated that consuming flavonoid-rich apples, particularly with the skin intact, leads to improved endothelial function. This improvement is characterized by enhanced flow-mediated dilation in individuals at risk for cardiovascular disease, attributed to increased NO bioavailability following both acute and four-week ingestion periods [144]. Similarly, findings by Ravn-Haren et al. indicate that whole fresh apples contribute to reduced total and LDL levels. In contrast, clear apple juice, which is devoid of water-soluble pectin and integral structural components, may lead to an elevation in these lipid parameters, underscoring the critical role of fiber in mediating hypocholesterolemic effects [145]. A study by Serra et al. supports these findings by reporting that apple varieties abundant in catechin, epicatechin, and procyanidin B1 are effective in lowering oxLDL and triglyceride concentrations [146]. Evidence from review studies indicates that whole-apple consumption significantly reduces total and low-density lipoprotein cholesterol levels, systolic blood pressure, pulse pressure, and plasma inflammatory cytokines, concurrently enhancing high-density lipoprotein cholesterol and endothelial function [147,148].

Moving beyond apples, other fruits such as avocados, grapes, pomegranates, blueberries, and citrus varieties also demonstrate distinct cardioprotective effects. Avocados contribute to cardiovascular health through their high content of monounsaturated fatty acids, fiber, and bioactive compounds, including polyphenols, carotenoids, and tocopherols [149]. Avocado intake has been shown to reduce TC, LDL, and triglycerides, with some studies also reporting increased HDL, largely mediated by oleic acid [149,150,151,152]. Additionally, it is demonstrated that avocado consumption improves postprandial glucose and insulin responses and enhances vascular reactivity markers such as flow-mediated vasodilation. Furthermore, avocado contains lipid derivatives that inhibit platelet aggregation and phytochemicals providing antioxidant and anti-inflammatory protection [149,153]. Experimental studies in animals support cardiovascular benefits, showing that avocado oil improves liver function and mitigates sucrose-induced metabolic disturbances [154,155].

Similarly, grapes-whether as whole fruit, seed, skin, or red wine extracts—are rich in polyphenols and exhibit broad cardioprotective effects. Leifert et al. reported that grape polyphenols such as resveratrol and anthocyanins act as antioxidants and anti-inflammatory agents, reducing LDL oxidation and modulating inflammatory pathways [107], while Luzak et al. showed enhancement of eNOS activity and improved vasodilation [156]. Zhao et al. observed reductions in blood pressure and improved lipid profiles, with lower TC, LDL, oxidized LDL, and higher HDL [141]. Shanmuganayagam et al. further demonstrated that grape-derived polymeric polygalloyl polyflavan-3-ols (PGPFs) inhibit platelet aggregation, contributing to anti-thrombotic effects [157].

Pomegranate is recognized for its potent atheroprotective and antihypertensive properties, largely due to its high antioxidant content, particularly punicalagin [63,141,158,159]. Pomegranate polyphenols scavenge free radicals, reduce oxidative stress, and modulate inflammatory pathways, thereby slowing atherosclerosis [63,158]. Pomegranate juice lowers blood pressure by inhibiting ACE activity [63,159,160], and extracts improve lipid metabolism, reduce aortic and coronary atherosclerosis, and enhance mitochondrial function through AMPK activation [63,158,159,161].

Blueberries, another flavonoid-rich fruit, also demonstrate protective cardiovascular properties. Rodríguez-Mateos et al. showed that blueberry supplementation improves endothelial function and lowers systolic blood pressure [162], while Zhao et al. observed enhanced lipid profiles, increased antioxidant enzyme activity, and anti-atherogenic effects, including reduced foam cell formation [141].

Other fruits, including mango, orange, and kiwifruit, further enrich the spectrum of cardiovascular benefits. Mango contains vitamin C, carotenoids, and polyphenols, and its constituent mangiferin reduces lipid peroxidation and preserves myocardial enzyme activities, protecting against myocardial infarction [63]. Oranges are particularly rich in vitamin C, folate, diverse polyphenols (primarily flavanones), as well as carotenoids (xanthophylls, cryptoxanthins, carotenes) and melatonin [92,163]. These bioactive compounds contribute to the modulation of oxidative and inflammatory pathways, thereby alleviating hyperglycemia and oxidative stress. Moreover, orange juice has been shown to inhibit key enzymes such as monoamine oxidase (MAO), phosphodiesterase (PDE), and ACE in heart tissue [63,164]. Kiwifruit, abundant in vitamins C and E and polyphenols, reduces platelet aggregation and plasma triglycerides upon daily consumption [165]. The soluble fiber naturally present in kiwifruit has been demonstrated to enhance dietary lipid absorption and reabsorption, thereby supporting lipid homeostasis. Moreover, studies focusing on whole kiwifruit have indicated additional beneficial effects on both blood lipid profiles and gastrointestinal function [63,166]. Taken together, these findings from experimental, clinical, and epidemiological studies-including large cohorts such as the Atherosclerosis Risk in Communities study (ARIC); The Prospective Epidemiological Study of Myocardial Infarction study (PRIME); Odyssey, and Nurses’ Health Study/Health Professionals’ Follow-up Study—demonstrate that fruit-based diets significantly reduce CVD risk, cardiovascular mortality, and incident coronary events. However, it is important to note that methodological variability, dietary assessment limitations, and confounding factors must be considered when interpreting these associations [92,141]. The protective effects of particular fruits, their compounds, and cardiovascular benefits are listed in Table 1.

### 5.2. Recommendations Regarding Fruit Intake

Fruits are an important part of the human diet, and their appropriate intake is recommended by health advisory organizations. The American Heart Association (AHA) advises two cups of fruits daily, and various forms are acceptable, including fresh, frozen, canned, or dried. The consumer needs to be aware that processed products may be less nutritious, and information on food labels should be analysed to make good health choices [189]. Similar recommendations regarding fruit intake can be found in guidelines by the Australian National Health and Medical Research Council (NHMRC)—min. daily intake of two servings of fruit for adults and children aged 9 and over [190], United States Departments of Agriculture (USDA) and Health and Human Services (HHS)—1.5 to 2 cups of fruit daily [191], and in the report by EAT Lancet Commission regarding Planetary Health Diet (PHD)—100–300 g of fruits daily [192]. However, most of the guidelines combine fruit and vegetable intake and present it as a single recommendation. WHO suggests consuming more than 400 g (5 portions, 80 g each) of fruits and vegetables per day to improve overall health and reduce the risk of certain non-communicable diseases (NCDs) [91]. Many European countries follow the “five a day” recommendation, where at least 5 portions of fruits and vegetables should be consumed. Whereas this recommendation can still be considered as a baseline minimum, some organizations are shifting towards higher values, i.e., >500 g [193]. Upward trend in recommended fruit and vegetable intake seems reasonable, as many studies confirm that risk reduction for cardiovascular disease, diabetes, obesity, and mortality is dose-dependent [194]. Lim et al. estimated that as many as 6.7 million deaths worldwide were attributed to insufficient fruit and vegetable intake in 2010 [195]. Nevertheless, according to the Centers for Disease Control and Prevention (CDC), only 12.3% of American adults met fruit intake recommendations in 2019. The suggestion regarding vegetable intake had even lower compliance and was equal to 10% [196]. The situation is similar in Europe, as only 12% Europeans were eating 5 portions of fruit and vegetables a day in 2021 [197]. Available data show that some Asian countries have better compliance; however, most Asians do not seem to meet the recommendations [198,199,200]. Public health efforts should be made to address low fruit and vegetable consumption worldwide. According to WHO, those actions should include: behavioural interventions, access to fruits and vegetables at low cost, and improvements in agricultural and food systems [201].

Moreover, the WHO emphasizes that consumption of fruits plays a key role in the prevention of CVD, as their consumption is strongly associated with reduced risk of both morbidity and mortality from heart and vascular diseases [91]. One of the nutritional models recommended by this organization is the Mediterranean diet. This dietary pattern is characterized by high consumption of plant-based products, including fruits, and according to the current data, it can be used in the prevention and control of non-communicable diseases [202].

## 6. Conclusions

Excessive intake of sugars has been consistently linked to adverse health outcomes, especially cardiovascular diseases; however, its evaluation must take into account the dietary source. Sugars naturally occurring in fruits do not exert the same unfavorable effects as refined sugars, due to the coexistence of bioactive compounds such as dietary fiber, polyphenols, antioxidants, and essential micronutrients, which collectively improve metabolism and exert anti-inflammatory effects. They are also inversely associated with cardiovascular morbidity and mortality. Furthermore, mounting evidence suggests that excessive added and free sugar consumption may promote hedonic eating and foster dependence on sweet taste; in contrast, fruit intake, while providing natural sweetness, contains compounds that slow sugar absorption and reduce reinforcement of sweet cravings.

Based on current data, fruits represent a functional food group that should be considered an integral component of cardiovascular disease prevention and overall metabolic health promotion. Future research should prioritize well-designed interventional clinical trials using fruit-rich diets to more comprehensively document their role in promoting cardiovascular health.

## Figures and Tables

**Table 1:** Fruits, their compounds, and their effects on CV activity.

Fruit	Compounds	CV Activity	References
Blueberry (*Vaccinium corymbosum*)	Anthocyanins; Flavonoids; Phenolic acids; Vitamin C, B complex, E, A; Carotenoids.	↑ Antioxidant capacity; ↓ Inflammation; Improved endothelial function—flow-mediated dilation.	Stull et al. [167]; Rodrigues-Mateos et al. [168]; Zuraini et al. [63]
Mango (*Mangifera indica*)	Vitamin C; Carotenoids; Polyphenols (maginferin); Flavonoids; Anthocyanins; Tannins; Phenolic acids; Coumarin.	Antioxidant; Anitinflammatory; Antiplatlet activity; Hypodipidemic: ↓ triglycerides, ↓ LDL ↓ TC, ↑ HDL; Enhanced endothelial function.	Alañón et al. [169]; Minniti et al. [170]; Castro et al. [171]; Lucas et al. [172]; Zuraini et al. [63]
Grape (*Vitis vinifera*)	Flavonoids (PGPFs); Anthocyanins (malvidin); Polyphenols (resveratrol).	Hypolipidemic ↓LDL, ↓TC; ↑ Antioxidant capacity; Improves blood pressure inhibiting platelet aggregation; Improved endothelial function.	Shanmuganayagam et al. [157]; Razavi et al. [173]; Borde et al. [174]; Freedman et al. [175]; Quintieri et al. [176]; Zuraini et al. [63].
Strawberry (*Fragaria* × *ananassa*)	Flavonoids; Anthocyanins; Phenolic acids; high content Vitamin C; rich Folate source.	↑ Antioxidant capacity; ↓ Inflammation; ↓ TC.	Miller et al. [177]; Basu et al. [178].
Apple (*Malus domestica*)	Flavonoids; Phenolic acids (chlorogenic acid and caffeic acid); Fiber (pectins); Phytosterols; Vitamin C; β-carotene	Hypolipidemic ↓ decreases LDL ↓TC; ↓Blood pressure; Antioxidant capacity; Antinflammatory; Enhances endothelial function.	Koutsos et al. [179]; Soleti et al. [180]; Liddle et al. [181]; Zuraini et al. [63].
Avocado (*Persea americana*)	Flavonoids; Phenolic acids; Carotenoids; Phytosterols; Fiber; Vitamin E; Monounsaturated Fatty Acids; Fatty alcohols	Antithrombotic; Antiplatelets; Antinflammatory; Hypolipidemic ↓ decreases LDL ↓ TC; Enhances endothelial function.	Park et al. [152]; Rodriguez-Sanchez et al. [153]; Olas et al. [182]; Zuraini et al. [63]
Pomegranate (*Punica granatum* L.)	Polyphenols; Flavonoids; Alkaloids; Vitamins; Sterols; Unsaturated fatty acids	Lipid Metabolism Regulation; ↓ Blood pressure; Protection of endothelial function; ↓ Oxidative stress; ↓ Inflammation	Haghighian et al. [161]; Sun et al. [159]; Hou C. et al. [183]; Zuraini et al. [63].
Orange (*Citrus sinensis*)	Vitamin C; Polyphenols (hesperidin, narirutin, naringin); Folate; Potassium	Lipid Metabolism Regulation; ↓ Blood pressure; ↓ Oxidative stress; ↓ Inflammation	Asgary et al. [184]; Miles et al. [185]; Zuraini et al. [63].
Kiwi (*Actinidia deliciosa*, *Actinidia chinensis*)	Vitamin C, K, E Folate; Polyphenols; Carotenoids; Potassium; Fiber	Lipid Metabolism Regulation; Antiplatelets; Antithrombotic; ↓ Blood pressure;	Duttaroy et al. [165]; Iwasawa et al. [186]; Karlsen et al. [187]; Stonehouse et al. [188]; Zuraini et al. [63].

Abbreviations: HDL–high-density lipoprotein cholesterol, LDL–low-density lipoprotein cholesterol, PGPFs—polygalloyl polyflavan-3-ols, TC–total cholesterol, ↓—indicates a decrease, ↑—indicates an increase.

## Data Availability

No new research data was created or analyzed in this study. Data sharing is not applicable to this article.

## Abbreviations

GLUT 4—glucose transporter 4, SGLT1—sodium-glucose cotransporter 1, T1R2/T1R3—sweet taste receptors.
